# Increased pain resilience among heavy metal music festival attendees

**DOI:** 10.1038/s41598-026-67389-x

**Published:** 2026-08-20

**Authors:** Lydia Schneider, Thomas H. Fritz

**Affiliations:** 1 Max Planck Institute for Human Cognitive and Brain Sciences, Stephanstrasse 1A, 04103 Leipzig, Germany; 2 Wilhelm- Wundt-Institute of Psychology, Clinical Psychology and Psychotherapy Unit, University of Leipzig, Neumarkt 9-19, 04109 Leipzig, Germany; 3 Institute for Psychoacoustics and Electronic Music (IPEM), Blandijnberg 2, Ghent, 9000 Belgium

**Keywords:** Neuroscience, Psychology, Psychology

## Abstract

This study investigated whether attending a metal music festival (which took place in August, 2023) could provide pain relief, particularly given that metal music is often associated with negative emotions such as anger. A between-group design compared a pre-festival group (*n* = 60) with a festival-attending group (*n* = 62) on measures of pain tolerance, pain sensitivity, pain unpleasantness, and heart rate variability (specifically, the standard deviation of normal-to-normal intervals, SDNN). Potential confounding variables, including general pain sensitivity, anticipatory joy, and alcohol consumption, were assessed. Results of a confirmatory analysis of the whole sample showed no significant differences in pain perception. Results of an exploratory analysis, which excluded participants exceeding a pre-defined threshold of heavy alcohol consumption, showed a significant difference in pain tolerance between the groups (F(1, 93) = 5.543, *p* = .021, Cohen’s d = 0.501). While pain sensitivity and pain unpleasantness did not differ between both groups, pain tolerance increased during the festival. These results may be interpreted as a proxy of increased resilience in participants during the festival and are discussed in terms of a stimulation of oxytocin release during an intense social music event. No evidence for improved emotional processing, as indexed by SDNN, was observed. The current study was a field study with a sample of metal music fans participating at the Wacken Open Air metal music festival. It is therefore not yet certain if the current results pertain to the Wacken Open Air experience, which is known among metal fans to be especially inclusive and friendly, for metal festivals in general, or for music festivals in general.

## Introduction

“People use music to satisfy a great breadth of needs and socio-cultural functions“^1^. Psychological functions of music listening have been described mainly with regard to three categories: arousal- and mood regulation, self-awareness and social relatedness^2^. These findings indicate that music listening serves primarily to support individuals’ psychological well-being. Interestingly, depending on the music genre music listening may not only include aesthetic enjoyment but also the confrontation with challenging expressed emotions, like sadness or anger.

### Music-induced emotional reactions

Music has the capacity to evoke a diverse spectrum of emotions through various perceptual and psychological mechanisms, and individuals’ emotional responses to music encompass multiple facets of emotional experience. Beyond the subjective feeling commonly associated with music listening, physiological responses can manifest as alterations in heart rate, breathing, skin temperature, and hormone release. Moreover, studies indicate that music listening engages brain areas implicated in emotional processing, such as the thalamus, hippocampus, amygdala, prefrontal cortex, orbitofrontal cortex, midbrain/periaqueductal gray (PAG), insula, and nucleus accumbens. Furthermore, music can shape a listener’s inclination towards certain actions, including the urge to move rhythmically and to engage in pro-social behaviors^3^. A study comparing the emotional impact of live versus recorded music across different age groups found, among other things, that the most pronounced positive emotional reactions occurred in individuals familiar with classical music when attending live performances^4^. Consequently, the researchers suggest that the emotional response to music depends on the characteristics of the audience and the mode of musical presentation (live or recorded;^4^. By integrating live music performances with social gatherings, music festivals can create particularly intense musical experiences that may amplify the psychological and physiological effects of music.

### Music-induced analgesia

Music as an emotion-inducer has been shown to also influence pain perception, especially by acting on the top-down modulation of pain via the descending pain modulatory system (DPMS), which is argued to relate to distraction, relaxation, positive emotion, or a combination of these mechanisms^5^. Music-induced analgesia refers to the reduced perception of pain through musical interventions, ranging from music medicine (music listening sessions administered by medical staff) to active music therapy^6^. The effects of music on pain have been researched across different types of pain, including acute, procedural and cancer/chronic pain^7^ and it has been shown that musical interventions decreased pain perception and reduced the required dose of anesthetics as well as opioids and non-opioids^7^. Underlying mechanisms of music-induced analgesia may also include different aspects of emotions, as described below.

Subjective feeling: The subjective feeling towards the pain experience may be influenced by changes in affect, such that music-induced analgesia may occur if musical stimuli are perceived as pleasant and subsequently result in a positive change of affect in the listener. It has been shown that only pleasant musical excerpts reduced pain intensity ratings and pain unpleasantness in participants, while unpleasant musical excerpts did not show such an effect^8^.

Psychophysiology: Music-induced analgesia may occur by lowering the arousal level of a person experiencing pain. Studies have shown that relaxing music reduced pain unpleasantness, while motivating music did not show such an effect. Such a change of arousal may also be related to a change of affect, as studies have shown that relaxing music not only led to decreased arousal levels but also led to reduced anxiety levels^9,10^.

Brain activation: Listening to pleasant music during a painful experience activates brain regions involved in emotion, cognition, and sound processing (e.g. certain limbic, frontal, and auditory areas), a finding absent when pain was experienced without music^11^. Furthermore, the pleasant music condition engaged brain regions associated with the descending pain modulation system, including the dorsolateral prefrontal cortex, periaqueductal gray, rostral ventromedial medulla, and dorsal gray matter of the spinal cord^11^. This initial study highlighted potential neural mechanisms underlying music-induced analgesia.

Emotion regulation: Music can regulate emotions through distraction or a shift in attention. For instance, music-induced analgesia might work by diverting attention from pain to the musical input, thus lessening the experience of pain. While emotional distractions like music have been shown to reduce pain more effectively than neutral distractions such as arithmetic tasks, their effectiveness is similar to other emotional distractions, for example humor when watching a cartoon^12^. Moreover, musical stimuli uniquely led to a greater sense of control over pain in experimental settings^12^. The emotional components of emotional expression and action tendency have not been directly studied in the context of music-induced analgesia. However, there is evidence that expressing one’s emotion verbally might be beneficial in terms of pain perception. This may be the case for emotions that occur most frequently when chronification of pain occurred, especially anger and anxiety. Studies have shown that anger expression among women with fibromyalgia predicted lower pain ratings as compared to anger inhibition^13^. Music has furthermore been suggested as an effective intervention for anger management, which may indirectly help moderate pain. Indeed, research suggests that listening to rather “angry” music when angry may represent an adaptive strategy of anger processing^14^. In addition, a study that utilized a repeated-measures design evaluated how exposure to heavy metal and classical music, moderated by individual preference, influenced the affective states of undergraduate participants. Note that the study did not apply an emotion induction paradigm (potentially resulting in less intense reported affective states). This study found that genre-specific stimuli generally induced distinct levels of arousal and hostility. Furthermore, regression analysis revealed that personal preference significantly moderated these effects, with metal-preferring individuals experiencing increased positive affect and reduced hostility following exposure. The findings underscore the critical role of listener preference as a predictor for mood induction^15^.

### Emotional processing through music

The idea that emotional processing can be guided through art dates back to Greek philosophy. In his *theory of catharsis*, Aristotle describes how tragedy or comedy through theatre could have a cathartic impact on the body (book Poetics, 350 B.C.^16^). Nowadays catharsis is defined as, “the process of releasing, and thereby providing relief from, strong or repressed emotions.”^17^. However, it is unknown how pain perception is altered through musical pieces that may serve as expressing one’s difficult emotions as compared to solely pleasant musical pieces inducing positive affect. Interestingly, „this idea of catharsis comes up a lot among metal fans and musicians. At its best, metal takes an unflinching look at life’s harsh realities, and provides a complex emotional response“^18^. While experimental studies often, perhaps rather casually, categorize metal music as “unpleasant” stimuli because they often express “negative” emotions, these investigations still provide valuable insights into the genre’s physiological and psychological impacts. For instance, a study by Ernberg et al.^19^ exploring the influence of background music on experimentally induced muscle pain in healthy women found that both “tuneless and noisy” black metal and “tuneful and soft” classical music had comparable effects on pain perception. Labbé et al.^20^ reported that college students who listened to different genres of music (self-selected relaxing music, classical music, heavy metal music) or silence after a stress test showed decreased anger as a consequence of all music conditions. Physiological measures showed that heart rate and respiration rate also decreased during all music conditions - here greater reductions in heart rate were observed in the self-selected and classical music conditions, while for the respiration rate greater reductions were observed in the classical and heavy metal music condition. With regard to anxiety and relaxation, self-selected and classical music reduced anxiety scores, while the heavy metal music selected for the experiment increased anxiety scores. For all conditions except the heavy metal music condition, an increase of feeling relaxed was reported. Physiological effects of different music genres in a sample of healthy participants showed beneficial effects on blood pressure and heart rate for both classical and heavy metal music^21^. A recent study investigated the stimulating effects of different music stimuli including metal music on six patients with disorders of consciousness^22^. Three different music stimuli were used: classical (Mozart), dodecaphonic (Schönberg), and heavy metal (Volbeat) music. The results showed that classical music in this experimental setup did not measurably change brain activity, while dodecaphonic music increased alpha and beta band activity in the right hemisphere. Perhaps notably, the heavy metal music used in this experiment can be argued to have induced the greatest stimulation of brain activity, such that as the authors write it “increased the delta and theta bands from the frontal lobes and the alpha and beta bands from most of the scalp“. No significant changes in synchronization were observed in either of the music conditions^22^. A recent randomized crossover trial, conducted at a heavy metal festival, investigated the impact of metal music on pain perception^23^. Forty-five participants underwent cold pressor tests while listening to either Slayer’s “Raining Blood” (thrash heavy metal music) or Enya’s “Orinoco Flow” (relaxing music). The study found that listening to thrash heavy metal music resulted in significantly lower pain intensity ratings (assessed with numeric rank scales of pain) compared to relaxing music, regardless of the listener’s personal fondness for the metal track. Additionally, participants who liked Enya actually reported higher pain levels during the test and higher breath alcohol levels were associated with decreased pain. Both music groups exhibited elevated heart rates that peaked shortly before maximum pain, though no statistically significant differences were found between the two music conditions or the consecutive test sessions.

### Aim of current study

Our aim was to explore how music, particularly intense musical experiences, might serve as a pain reliever. Specifically we investigated music-induced analgesia at the Wacken Open Air Festival for heavy metal music. Music has been widely recognized as a human universal^24^, which probably also relates to a variety of functions that music has for the individual. Avoiding pain and approaching pleasure have been argued to be key components of human motivation^25^. That people go to music festivals to see their favorite bands is clearly linked to an „approaching pleasure“ behavior. However, it is unknown if this is also linked to a reduction in pain perception. Furthermore, we wanted to study if an intense music experience at a music festival may also alter the quality of emotional processing in terms of changes of heart rate variability measures. The context of this study was chosen in order to allow for studying human pain perception in a naturalistic context (music festival), also because live music performances have been shown to often evoke stronger emotional responses^26^ than music in laboratory experimental set-ups. Music festivals are social rituals that can attract vast numbers of participants over a duration of several days. Here we investigated pain threshold before and during a music festival as an indirect indicator of endorphin levels to gain more insight in why going to festivals may be motivating on a physiological level.

### Hypotheses

The study aims at investigating the effects of a metal music festival where participants were exposed to angry music on pain perception and will test the following hypotheses: H1 (confirmative): Pain perception (including pain sensitivity and pain unpleasantness) is reduced in metal music listeners during a heavy metal music festival as compared to pain perception of metal music listeners on the day before the festival (baseline). H2 (explorative): emotional processing in terms of heart rate variability is enhanced during the music festival as compared to before the festival.

## Methods

### Participants

A total of 122 participants were recruited for the current field study between July, 31st and August, 6th, 2023 during a German metal music festival (therefore participants were mainly Germans). Sixty participants were recruited on the festival site of the Wacken Open Air heavy metal music festival on the day before the festival started in order to perform baseline measures, and sixty-two participants were recruited on the 3rd and 4th day of the festival. A priori computation of the required sample size was performed using G*power (effect size d = 0.5; alpha-error probability = 0.05; power = 0.8) resulting in a required sample size of 64 participants per group (in total 128 participants). For a detailed description of both participant groups (before the festival group vs. during the festival group) see the table below (Table 1). Informed consent was obtained from each participant before the study started. Any individual displaying observable signs of intoxication was strictly excluded prior to recruitment and consent. As such, all participants included in the study possessed full cognitive capacity to provide informed consent. The study adhered to the guidelines of the Declaration of Helsinki and was approved by the ethics committee of the University of Leipzig, Germany. This study`s design and hypotheses were pre-registered prior to any analysis of collected data at *OSF.io* and can be assessed via the following registration link: 10.17605/OSF.IO/XKGSM.

**Table 1:** Participant characteristics for both groups.

Characteristic	Participant group = 0 (Before the festival; *n* = 60)	Participant group = 1 (During the festival; *n* = 62)	*P* value
Age (M, SD)	33.73, 10.55	35.95, 9.71	> 0.05
Body Mass Index (M, SD)	28.38, 5.36	28.79, 5.35	> 0.05
PSQ-minor (Mdn, Var)	2.28, 1.67	2.42, 1.04	> 0.05
PSQ-moderate (Mdn, Var)	4.57, 2.22	4.57, 1.81	> 0.05
PSQ-total (Mdn, Var)	3.93, 1.89	3.64, 1.30	> 0.05
Anticipatory joy (Mdn, Var)	8.25, 3.33	9.00, 4.30	> 0.05
AUDIT (Mdn, Var)	4.00, 6.92	4.00, 4.83	> 0.05
Alcohol consumption (M, SD)	3.00, 2.39	3.34, 3.02	> 0.05
Gender (male: female: nonbinary)	43:17:0	46:16:0	> 0.05
Handedness (right: left: both)	54:6:0	56:3:2	> 0.05
Musician (no: yes)	53:7	61:1	> 0.05
Music instrument (no: yes)	40:20	43:19	> 0.05
Chronic pain condition (no: yes)	48:12	50:12	> 0.05

Note. The table displays participant characteristics for both groups: age (in years); body mass index; pain sensitivity questionnaire (PSQ) scores for the sub-scales minor, moderate and total; joy before/during the festival on a 100 mm visual analog scale; AUDIT-C alcohol consumption questionnaire; alcohol consumption on the day of the study; ratios for gender, handedness, musicians and music instrument playing, chronic pain conditions. Note, if ratios do not add up to the sample size of the group, this was due to missing responses in the corresponding questionnaire.

### Experimental design

**Fig. 1:**
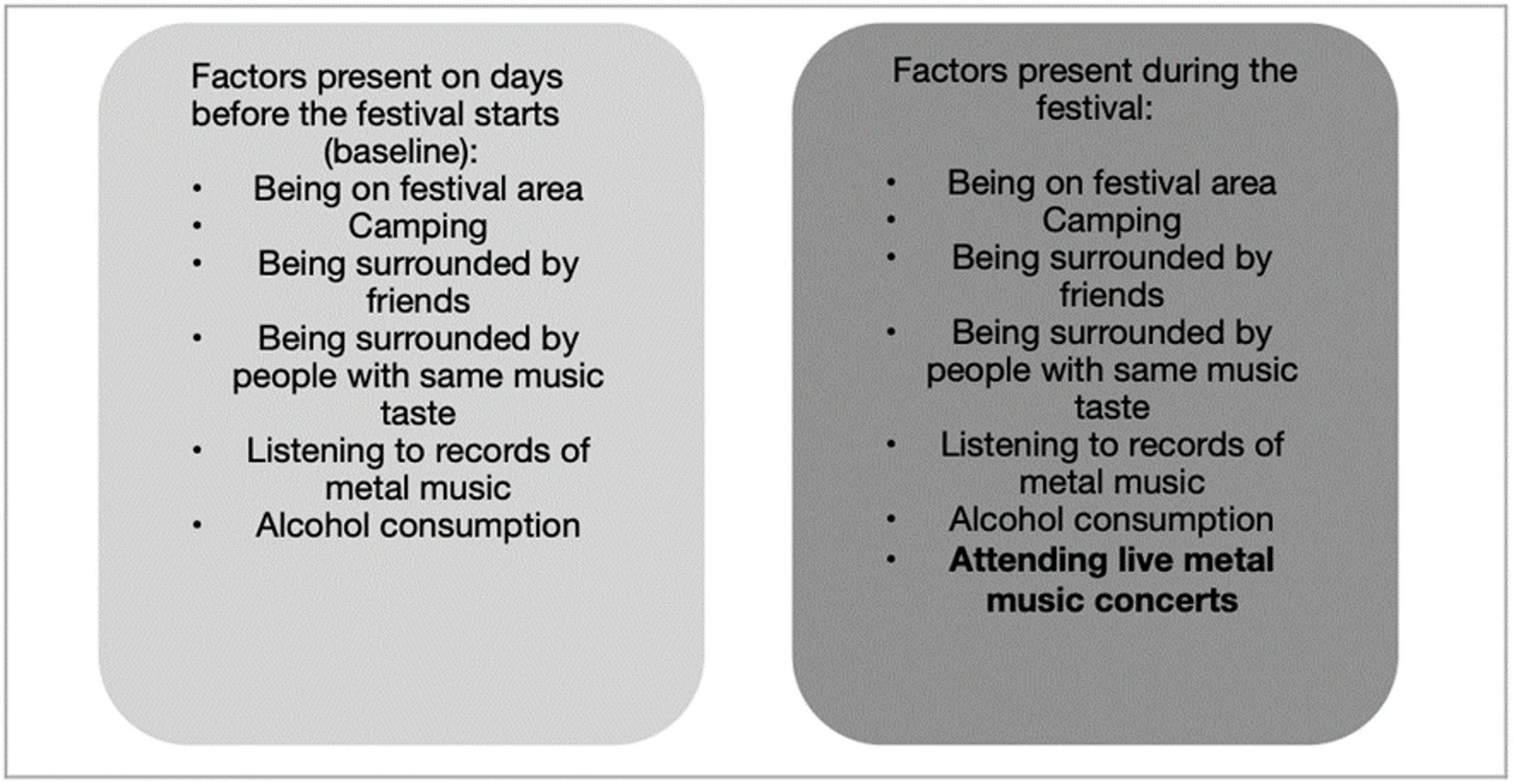
Description of external factors present before the festival started and during the festival. Note. This figure shows a schematic depiction of external factors present before the festival starts compared to external factors present during the festival.

A between-group design was used in which 60 participants, were recruited for the study on the festival area one day before the festival started (group 0 = baseline before the festival group) and 62 participants were recruited during the 3rd and 4th day of the festival (group 1 = during festival group; see Fig. 1). The study was set up in a festival tent opposed to a central festival clothing merchandise booth. This ensured that a majority of festival visitors would pass by, got to know the study and could decide if they wanted to participate. Both participant groups first underwent a resting electrocardiogram recording (duration of five minutes), while standing near a table and filling out questionnaires on demographics, then an acute cold pressor pain task was performed. Standardized psychological questionnaires were used to assess possible confounding variables: general pain sensitivity, alcohol consumption in general and on the day of participating in the study, pre-existing chronic pain conditions, and amount of listening to metal music during daily routine.

### Assessment of pain sensitivity, pain tolerance and pain unpleasantness

During the cold pressor task (CPT)^27^, participants are asked to place their non-dominant hand and forearm into cold water for as long as they can tolerate it. The time from placement of the hand and forearm into the water until perceived pain is reported was used as a measure of pain sensitivity. The time from reported perceived pain until withdrawal was used as a measure of pain tolerance. Water entry, time point of reported perceived pain and withdrawal times were recorded with a stopwatch (values rounded to the nearest hundredth of a second). Accuracy was facilitated by a mechanical lever in the water that responded to the weight of a participant’s hand by producing a clear visual signal for the experimenter. To prevent tissue damage, the CPT was always stopped after four minutes maximum. The test apparatus itself consisted of a water tank with a built-in refrigeration unit that was adjusted to cool the water to 2 °C. Water temperature was continuously controlled before each cold pressor task performance to ensure a similar temperature for each trial and to control for the temperature variations that occur as a result of the cooling process (which uses a thermostat to switch water cooling on and off) as well as changes caused by the procedure itself (e.g., warming of the water caused by the temperature and surface area of the submerged limb). A visual analog scale of pain unpleasantness (0–100 mm) was used to assess how unpleasant participants perceived the pain during the cold pressor task (CPT) and immediately after finishing the CPT.

### Assessment of heart rate variability

Heart rate variability (HRV) is a commonly used measure of stress reactivity which has been argued to relate to quality of emotional processing^28^. The physiological data was acquired by using BioHarness 3.0 devices (Zephyr Technology Corporation, Annapolis, MD, USA). At the beginning of the study, the chest strap with sensor was placed at the lower part of the chest, as indicated in the BioHarness 3.0 user manual. The device stores data, including electrocardiograms (ECG), heart rate (HR), and RR intervals. The BioHarness Log Downloader software (Zephyr Technology Corporation, Annapolis, MD, USA) was used to download the RR interval data. HRV data analysis of time domain measures such as R-R interval, standard deviation of R-R interval (SDNN), root mean square of successive differences (RMSSD) and mean heart rate (HR) were obtained by using the Kubios software version 1.1 (Bio-signal Analysis Group, Kuopio, Finland). The ECG was recorded for three minutes for each participant, allowing for analyzing data of two minutes duration. The length of the ECG recording was chosen based on the findings by Munoz et al. (2015), showing that it is reliable to use recordings of 120s to obtain accurate measures of SDNN and RMSSD in the time domain of HRV measures.

### Assessment of general pain sensitivity

Pain-Sensitivity-Questionnaire (PSQ) – assesses individual differences in general pain sensitivity in everyday life. The PSQ consists of two subscales besides the PSQ total score, the PSQ-minor (sample item: „Imagine your muscles are slightly sore as the result of physical activity“.) and the PSQ-moderate scale (sample item: „Imagine you bump your elbow on the edge of a table.“), which divide the items into those describing mildly painful (mean rating < 4) and moderately painful situations (mean rating 4–6). PSQ scores have been shown to have high internal consistency and are independent of age and gender^29^.

### Assessment of anticipatory joy before and during the festival

A 10-point scale (0 = not at all, 10 = extremely joyful) was used to verbally assess the current level of anticipatory joy of the participants.

### Assessment of general alcohol consumption behavior and current alcohol consumption behavior

The Alcohol Use Disorders Identification Test-Concise (AUDIT-C;^30^ is a brief alcohol screening instrument that reliably screens for active alcohol abuse or dependence behavior. The AUDIT-C has three questions (“How often do you have a drink containing alcohol?”, “How many units of alcohol do you drink on a typical day when you are drinking?”, „How often do you have six or more drinks on one occasion?”), and can reach a total score from 0 to 12 points. Each AUDIT-C question has five answer choices valued from 0 to 4 points. Generally the higher the score, the more likely it is that a person displays risky drinking behavior. The questionnaire was included as some studies have shown that individuals with alcohol use disorder and moderate/heavy drinkers have a reduced heart rate variability as compared to healthy individuals^31^. In addition to the AUDIT-C we added a question addressing how many alcoholic beverages were consumed on the day when the testing took place: „How many standard drinks containing alcohol did you have today?“ with the same 5 answer choices as suggested by the AUDIT-C. This question was added, as a systematic review and meta-analysis by Thompson et al.^32^ on the effects of alcohol on pain perception reported significant effects of acute alcohol consumption on pain sensitivity (pain threshold) and pain intensity ratings of participants^32^. Participants showed a heightened pain threshold (lower pain sensitivity) after consumption of alcohol (small effect) and reported reduced intensity ratings of pain (moderate to large effect). Furthermore, acute alcohol consumption has been shown to reduce resting heart rate variability (HRV) in healthy subjects^31^.

### Data analysis

All data were first assessed for normality using skewness and kurtosis. In cases where assumptions of normality were violated, appropriate data transformations (e.g., log transformation) were employed to approach a normal distribution. If a normal distribution was not achieved despite these transformations, non-parametric equivalent tests were utilized for further analyses. First, descriptive statistics characterized both groups in terms of age, gender, handedness, body mass index (BMI), alcohol consumption, music preferences, general pain sensitivity, and water temperature. Subsequently, independent samples t-tests were conducted to ensure that both groups did not differ significantly on these baseline variables. If any variable differed significantly, it was included as a covariate in a subsequent analysis of covariance (ANCOVA). Secondly, an ANCOVA was performed on differences in acute pain perception (pain sensitivity, pain tolerance, and pain unpleasantness) between groups to test the confirmatory hypothesis. An additional exploratory analysis of pain perception was performed excluding heavy drinkers. Furthermore, an independent samples t-test was used to exploratively test for differences in heart rate variability (HRV) between groups, and to determine whether HRV became more homogeneous within the festival group compared to the baseline group. Finally, a correlation was performed to analyze the relationship between changes in pain perception and changes in heart rate variability.

## Results

Preliminary analysis of participant characteristics was performed revealing no significant differences between both groups regarding the following characteristics (see Table 1): age, body mass index, general pain sensitivity (PSQ scores), joy before/during the festival, scores of the AUDIT-C alcohol consumption questionnaire, amount of alcohol consumption on the day of testing, gender, handedness, musicians/music instrument playing, and the presence of chronic pain conditions.

The study aimed at investigating the effects of a music festival promoting rather angry metal music on pain perception and tested the following confirmative hypothesis (H1): Pain perception (including pain sensitivity and pain unpleasantness) is reduced in metal music listeners during a heavy metal music festival as compared to pain perception of metal music listeners before the festival (baseline). Log-transformations were used to normalize the data of pain sensitivity and pain tolerance. An analysis of covariance (ANCOVA) was performed in order to control for water temperature as a covariate as water temperature differed between both groups. The ANCOVA showed no significant differences in pain tolerance, pain sensitivity and pain unpleasantness between both groups.

**Fig. 2:**
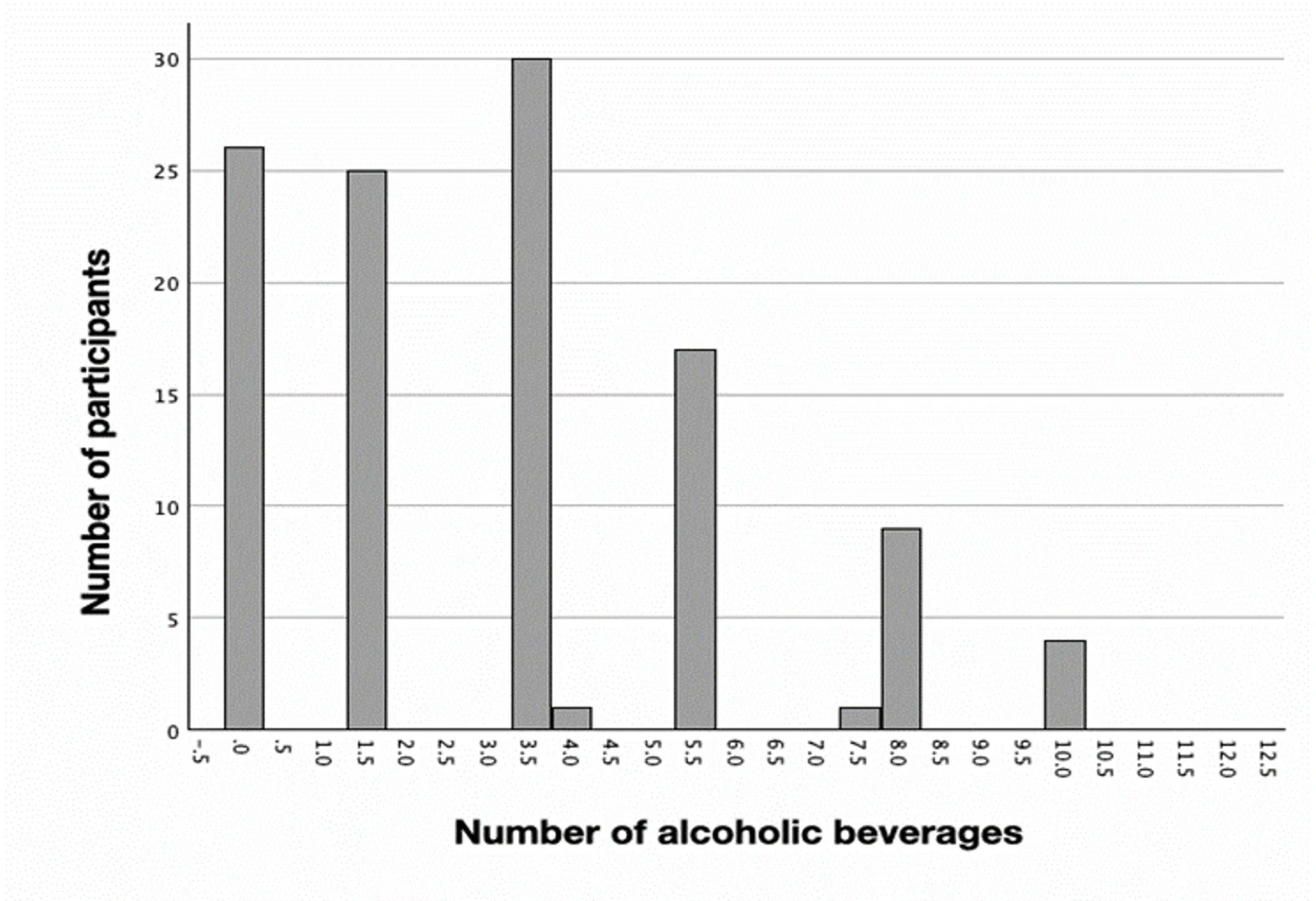
Frequency of number of alcoholic beverages consumed on the day of testing. Note. A histogram of frequency of number of alcoholic beverages consumed on the day of testing for the whole participant sample (data available for *n* = 113).

Based on the visualization of descriptive statistics showing that a minority of participants (fourteen participants) were displaying heavy drinking behaviour on the day of testing (see Fig. 2) and with respect to previous meta-analytic research showing that higher blood alcohol concentrations were associated with lower pain intensity ratings^32^, we decided to additionally investigate if removing participants who drank excessively during the day of testing (defined as more than six drinks on one occasion by the standardized AUDIT-C questionnaire) might show effects on pain perception that are otherwise masked by heavy alcohol consumption effects on participants.

**Fig. 3:**
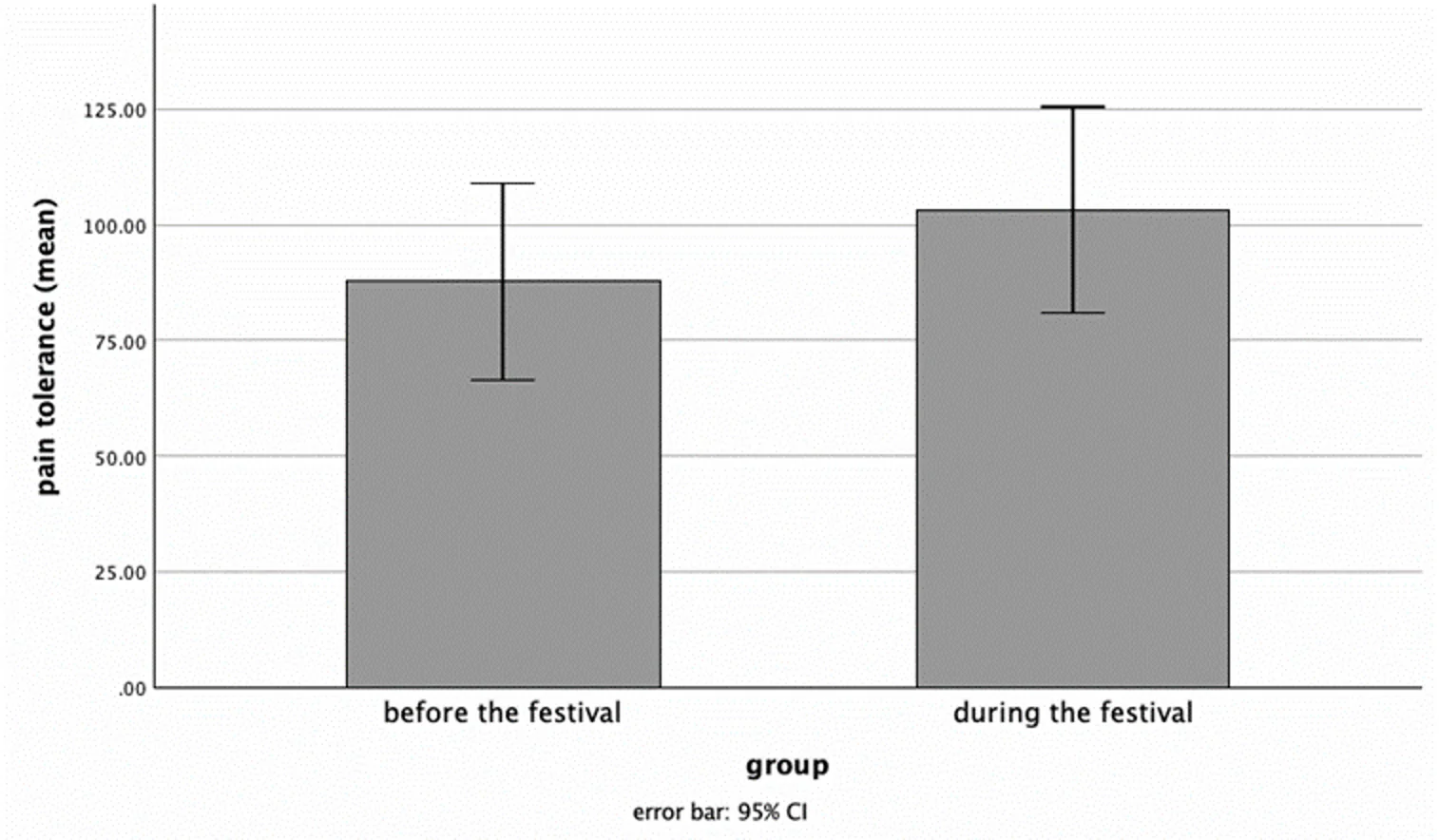
Differences in pain tolerance between both groups including all participants. Note. A histogram of mean pain tolerance in seconds for both groups, before the festival group and during the festival group. Error bars indicate the 95% confidence interval.

**Fig. 4:**
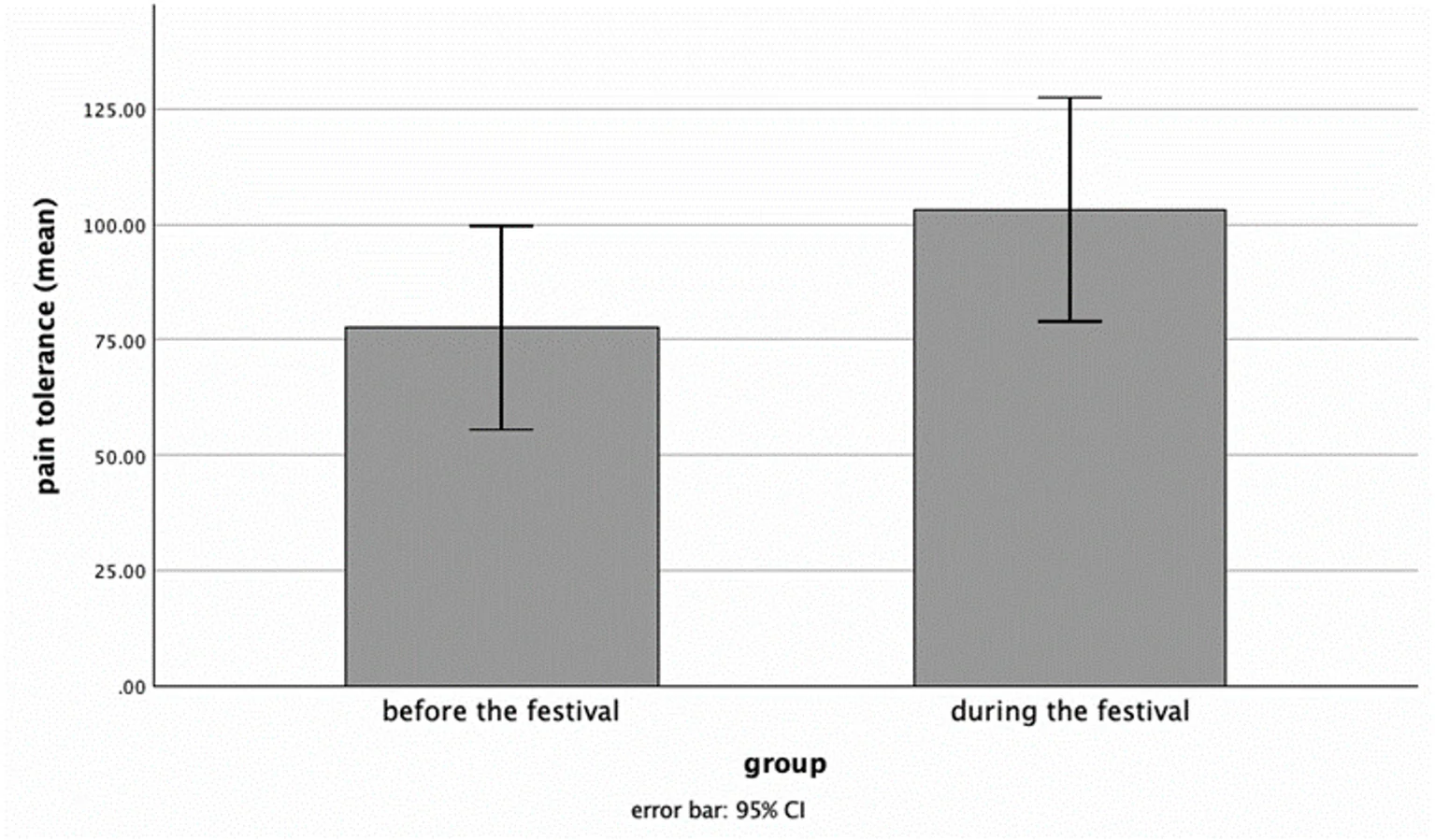
Differences in pain tolerance between both groups after excluding heavy drinkers. Note. A histogram of mean pain tolerance in seconds for both groups, before the festival group and during the festival group after excluding those participants who reported very high alcohol consumption on the day of testing (six and more alcoholic beverages). Error bars indicate the 95% confidence interval.

An additional exploratory analysis of pain perception excluding heavy drinkers was performed. Results of an ANCOVA (with the covariate: water temperature) showed that group had a significant effect on pain tolerance (*n* = 97), such that the baseline group (*n* = 48; log mean = 1.54, log SD = 0.72) had lower pain tolerance levels as compared to the festival group (*n* = 49, log mean = 1.79, log SD = 0.63) with F(1,93) = 5.543, *p* = .021, 95% CI [-0.576, − 0.049] and partial eta squared of 0.056 and an effect size Cohen´s d of 0.501 (medium effect, with a post-hoc achieved power of 0.79). Pain sensitivity did not differ significantly between baseline group (*n* = 48; log mean = 1.36; log SD = 0.31) and festival group (*n* = 49; log mean = 1.26; log SD = 0.31). Unpleasantness of pain did not differ significantly between both groups (Figs. 3, 4).

Furthermore, we investigated the following hypothesis (H2; explorative): quality of emotional processing is enhanced during the music festival as compared to before the festival. The HRV data was log-transformed to normalize the data. Results showed that heart rate variability (SDNN) did not differ significantly between both groups (see Table 2). There was no difference in heart rate variability before and during the festival. Additionally, heart rate did not differ between both groups (see Table 2).

**Table 2:** Measurements of heart rate variability for both groups.

	Before the festival - group M (SD)	During the festival - group M (SD)
HR (log)	1.96 (0.06)	1.95 (0.06)
SDNN (log)	1.51 (0.21)	1.54 (0.20)
RMSSD (log)	1.40 (0.28)	1.37 (0.25)

Note. Log-transformed means and standard deviations for heart rate (HR), standard deviation of NN intervals (SDNN) and root mean square of successive RR interval differences (RMSSD) for both groups, before the festival group (*n* = 42) and during the festival group (*n* = 50).

**Fig. 5:**
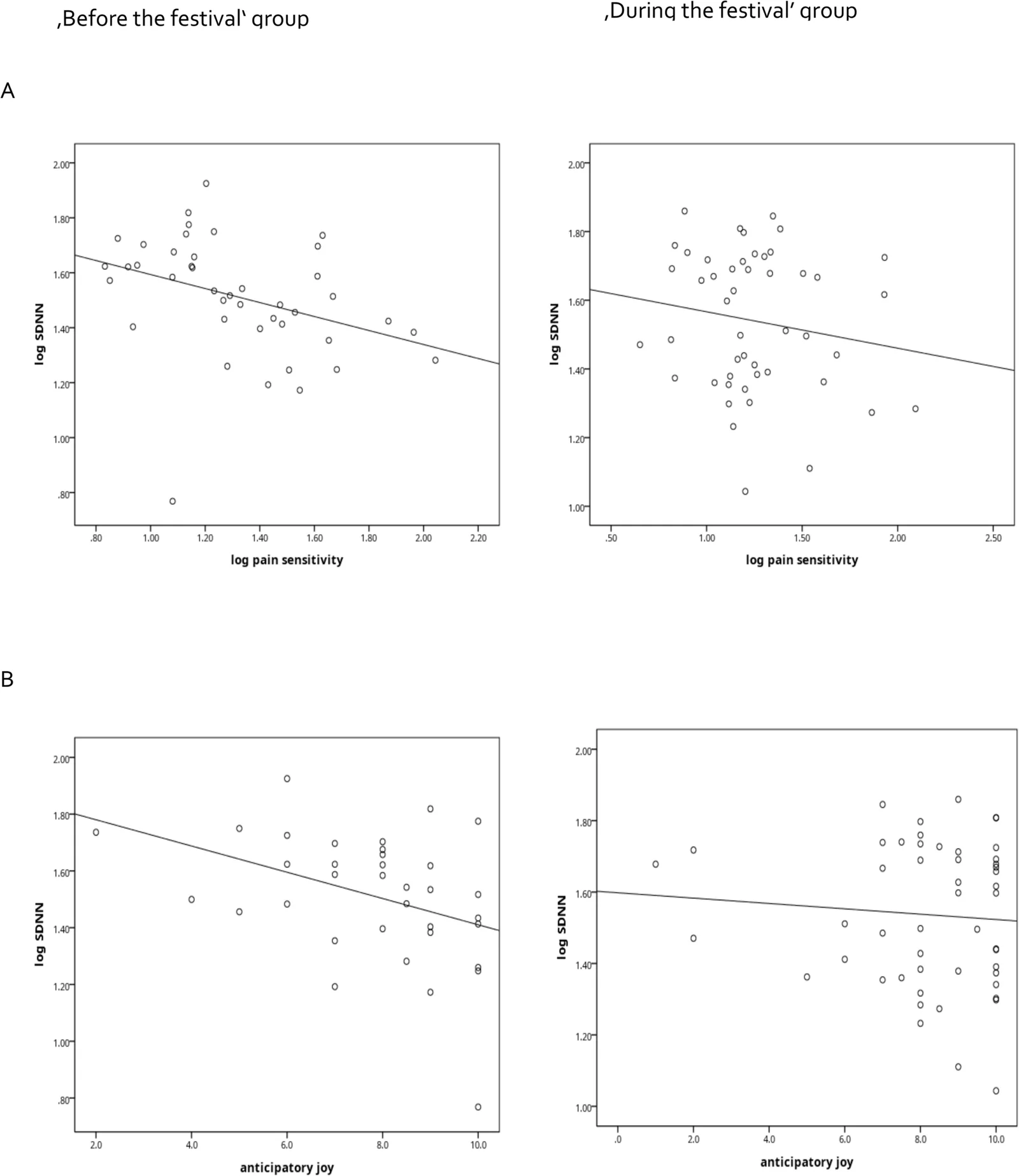
Correlations of heart rate variability, pain and anticipatory joy for both groups. Note. This figure displays (**A**) a significant negative correlation of standard deviation of NN intervals (SDNN) and pain sensitivity for the baseline group (before the festival started; *r* = − .359, *p* = .020, *n* = 42) and a non-significant correlation of SDNN and pain sensitivity for the festival group (*r* = − .160, *p* = .277, *n* = 48). (**B**) a significant negative correlation of SDNN and anticipatory joy for the baseline group (*r* = − .404, *p* = .016) and a non-significant correlation of SDNN and anticipatory joy for the festival group (*r* = − .065, *p* = .654, *n* = 50).

**Fig. 6:**
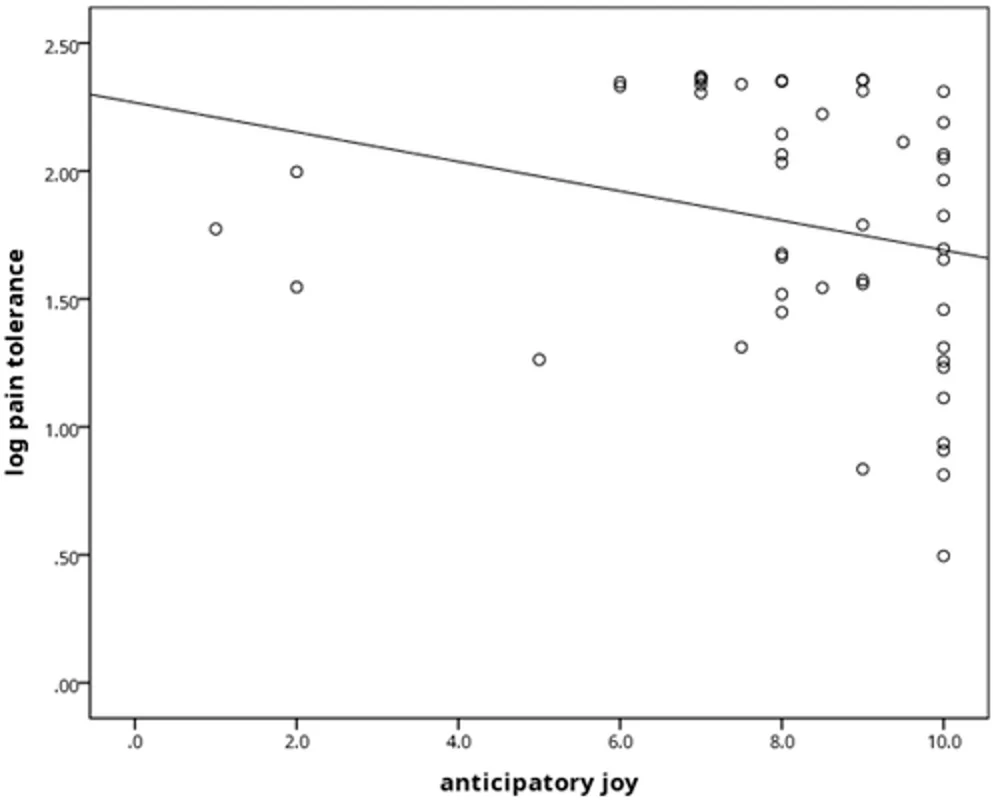
Correlation of pain tolerance and anticipatory joy for the festival group. Note. This figure displays a significant negative correlation of pain tolerance and anticipatory joy for the festival group (*r* = − .416, *p* = .003, *n* = 49).

Interestingly, the influence of heart rate variability on pain parameters seems different for both groups. At baseline before the festival started, participants heart rate variability (SDNN) correlated significantly negatively with pain sensitivity (Pearson`s correlation: logSDNN and log10pain sensitivity (*r* = − .359, *p* = .020, *n* = 42)). Such an influence of heart rate variability (SDNN) on pain sensitivity could not be observed for the festival group (*r* = − .160, *p* = .277, *n* = 48). Furthermore, ratings of anticipatory joy correlated negatively with heart rate variability (Spearman`s correlation: joy and log SDNN: *r* = − .404, *p* = .016) in the baseline group, while in the festival group anticipatory joy did not correlate significantly with heart rate variability (*r* = − .065, *p* = .654, *n* = 50). However, in the festival group anticipatory joy correlated significantly negatively with pain tolerance (Spearman’s correlation: anticipatory joy and log10pain tolerance (*r* = − .416, *p* = .003, *n* = 49)). The datasets generated during the current study are available from the corresponding author on reasonable request (Figs. 5, 6).

## Discussion

The present study investigated music-induced analgesia through an intense music experience, the Wacken Open Air Festival promoting heavy metal music, a music genre known to predominantly express unpleasant emotions like anger. The current study aimed at increasing our understanding of how analgesic musical effects may be essential to such modern day musical rituals and could thus perhaps even contribute to why music is a human universal^24^.

While a previous experiment shows that metal listeners while in the process of listening to metal music perceive pain as less intense compared to listening to relaxing music^23^, we here show in an exploratory analysis an increase in pain tolerance during the course of a metal festival as compared to before the festival (when participants with heavy alcohol consumption on the day of testing were excluded). Such effects were not observed with regard to pain sensitivity and unpleasantness of pain nor when the whole sample was analyzed. The results also seem to suggest that changes in pain tolerance seem not to be explained by increased pleasantness such that anticipatory joy did not differ between groups and furthermore correlated negatively with pain tolerance in the festival group. Also, they seem not to be explained by an enhanced emotional processing in terms of increased heart rate variability. Participants perceived their pain during the festival not as less unpleasant or were less sensitive in perceiving it, but they could endure it for a longer time. This seems to indicate that participants got more resilient, which means that they might have to a greater degree accepted feelings of unpleasantness towards the pain experience and focused on the factors which are in their own control (self-efficacy), e.g. time until they actually act upon their feelings of unpleasantness by removing their hand and forearm from the ice-cold water. The concept of resilience is defined by the American Psychological Association^33^ as „the process and outcome of successfully adapting to difficult or challenging life experiences, especially through mental, emotional, and behavioral flexibility and adjustment to external and internal demands.“ In the context of our study, pain tolerance might accordingly be interpreted as a proxy of increased resilience during the metal music festival. However, we did not implement a validated resilience questionnaire (e.g., the Brief Resilience Scale or the Connor-Davidson Resilience Scale) and therefore resilience was not directly measured in the current study but pain tolerance was used as a proxy for resilience. A recent study by Buckingham and Richardson^34^ demonstrated the impact of resilience on pain perception. Here, different factors of resilience were investigated (optimism and grit) and findings showed that resilience (and its constituent factors) was positively related to pain tolerance (and time to pain threshold), but not to ratings of pain severity. Authors argue that investigating an individual’s resilience may be important when it comes to acute pain perception, as pain represents an adverse event. Interestingly, resilience towards pain has also implications for chronic pain conditions. A clinical study including over 400 patients with chronic spinal pain demonstrated that accepting one’s pain seems to lead to a reduced pain perception and this acceptance of pain is strongly influenced by how resilient an individual is^35^. Therefore, a music festival increasing pain resilience at least temporarily could perhaps even to some degree help to counteract chronic pain or developing chronic pain.

A possible increase in resilience during the festival might also be indicated by the negative correlation of anticipatory joy and pain tolerance in the festival group, as this shows that positive feelings did not moderate pain tolerance but rather the contrary. This underlines the hypothesis that accepting unpleasant emotional states or difficult situations plays a central role in being resilient and in tolerating pain instead of masking unpleasant emotions with positive emotions. It may be worth noting that this aligns with common themes in metal music, which often expresses difficult emotions and life challenges avoiding attempts to mask them with happiness. Combining such expression of negative emotionality with an energetic and activating soundscape may foster self-efficacy, an important component of resilience.

The interpretation of increased pain tolerance in the context of resilience could also explain from a psychological perspective why when individuals with high alcohol consumption were included in the data analysis no effect on pain tolerance was observed. Alcohol as an external factor could have lowered experiences of being resilient as it diminishes experiences of self-efficacy and being in control over one’s own behavior. It might be argued that a festival lasting only a few days is insufficient to impact resilience. However, a rather intense musical experience (live music conveying emotions, being with friends, facing challenges during festival etc.) might serve as an extraordinary event, which impacts resilience in such a way that its influence on pain perception is measurable after three to four days. There is evidence that resilience can be strengthened by short-term intense interventions, such as intensive meditation or willpower strengthening training^36,37^. More specifically, a randomized-controlled trial comparing the effects of four days of intensive meditation versus four days of relaxation (control group) showed a significant increase in mindfulness and resilience for both groups immediately after the intervention days as compared to baseline measures^36^. Given the importance of resilience in the context of pain management, a future study on effects of music experiences and pain perception should include a specific assessment of resilience in participants. Furthermore, other factors beyond resilience could have led to an increased pain tolerance as observed in our study. The CPT measure of pain is a behavioural measure which may have been influenced by a wide range of factors specific to the festival environment including psychological aspects like participants being more socially motivated to perform well during the pain task or changes in participants physiology during the festival (e.g., elevated cortisol or endorphin levels from physical activity and excitement or oxytocin release during intense social interactions during concerts).

The results of the current study may support the „oxytocin-hypothesis of music“. Recent research has suggested that music stimulates endogenous oxytocin release that plays a prominent role in dopaminergic and opioidergic regulation during reward processing, enhancing sensitivity to musical rewards within a social setting^38^. A music festival context as researched in the present study might have stimulated endogenous oxytocin release in participants too, and may have subsequently influenced pain perception. While some reviews indicate that oxytocin reduces pain sensitivity^39,40^, meta-analyses on exogenous administration remain inconclusive. In this festival context, while live music likely boosted oxytocin-induced analgesia, acute alcohol consumption may have inhibited this release^41^. However, since neurohormonal markers were not measured, these physiological mechanisms remain speculative and could involve a broader confluence of catecholamines.

### Limitations

The study sample is representative of the metal subculture as participants were recruited as part of a field study (the study took place on the metal music festival grounds). The diversity of the study sample is therefore representative of typical characteristics of this subculture (e.g. mostly male participants, certain age range) and also in terms of cultural background (mostly German sample), which may limit generalizability of the study findings. In other words, it is not certain if the current results are only true for the Wacken Open Air, which is known among metal fans to be especially inclusive and friendly, for metal festivals in general, or for music festivals in general. In the current study a subjective assessment tool of alcohol consumption was used, which was readily accepted by participants but their answers may have been biased by social desirability or individual memory of alcoholic beverages consumed during the testing day, and objective assessments like measuring breath alcohol concentration could offer a more precise tool. We limited the assessment of emotions to anticipatory joy in order to reduce the total time of testing per participant. However, this limits the insights into emotional changes during the festival. Whether the observed effects were due to participating at a festival in general or specifically at a heavy metal music festival cannot be differentiated at this time.

### Constraints on generality

#### Participants

The sample of participants in our study is representative of a broader target population, which is characterized by a comparable cultural background, age, gender ratio (mainly male participants), body mass index, general pain sensitivity and general alcohol consumption behavior. For different cultural backgrounds and age groups it would be interesting to investigate if similar effects can be observed.

Materials/Stimuli: The pain task used in the current study generalizes to other pain tasks that measure cold pain. Other experimental pain tasks that measure e.g. heat or pressure pain may display different results^42^, but were not investigated in the current study. As noted in the section *limitations* of the study, objective measurements of alcohol consumption instead of subjective measurements (questionnaire) as used in our study, may result in a more precise investigation of the influence of alcohol on pain. The investigated musical stimuli (live metal music concerts) can be generalized only to festivals of the same music genre.

#### Procedures

Regarding the procedure of the study, it is important that participants of the festival were already present at the festival area a couple of days before the concerts started. This allowed for a comparable environmental context (camping, surrounded by friends, consumption of alcohol and listening to music) except there were no live music concerts during baseline assessment. If this procedure cannot be followed, it will be difficult to control for confounding variables and thus may result in different findings.

Historical/temporal specificity: Differences in the historical/temporal context might affect the characteristics of the participant sample, e.g. in terms of age or gender ratio, depending on changes in popularity of the specific music genre of metal music in the general population and/or changes in diversity in the metal music community (e.g. more FLINTA* metal bands on stage).

### Summary

In summary, the current study investigated whether attending a heavy metal music festival could have analgesic effects. Using a between-group design, the study compared a pre-festival group (*n* = 60) with a festival-attending group (*n* = 62) on measures of pain tolerance, pain sensitivity, pain unpleasantness, and heart rate variability (SDNN). After excluding participants with heavy alcohol consumption, results showed a significant increase in pain tolerance during the festival, suggesting enhanced resilience to pain. This effect was not observed for pain sensitivity or unpleasantness. No evidence for improved emotional processing, as indexed by heart rate variability, was found. The increased pain tolerance may be linked to oxytocin release during intense social music events.

## Data Availability

Data will be shared upon reasonable request.
